# The association between oral hygiene and periodontitis: a systematic review and meta‐analysis

**DOI:** 10.1111/idj.12317

**Published:** 2017-06-23

**Authors:** Attawood Lertpimonchai, Sasivimol Rattanasiri, Sakda Arj‐Ong Vallibhakara, John Attia, Ammarin Thakkinstian

**Affiliations:** ^1^ Section for Clinical Epidemiology and Biostatistics Faculty of Medicine Ramathibodi Hospital Mahidol University Bangkok Thailand; ^2^ Department of Periodontology Faculty of Dentistry Chulalongkorn University Bangkok Thailand; ^3^ School of Medicine and Public Health Centre for Clinical Epidemiology and Biostatistics University of Newcastle Newcastle NSW Australia; ^4^ Hunter Medical Research Institute Newcastle NSW Australia

**Keywords:** Meta‐analysis, oral hygiene, periodontitis, risk factor, systematic review

## Abstract

**Objective:**

Dental plaque accumulation and inadequate personal oral hygiene (OH) are known major risk factors of periodontitis. Nevertheless, the magnitude of their effects has not yet been the subject of a meta‐analysis.

**Material and methods:**

The Medline and Scopus databases were searched up to May 2016. Observational studies were eligible if they assessed associations between OH and periodontitis in adult subjects. A multivariate random‐effects meta‐analysis was used to pool the effects of fair/poor OH *versus* good OH on periodontitis across studies. The associations between oral care habits and periodontitis were also assessed.

**Results:**

A total of 50 studies were eligible; 15 were used for pooling the effect of fair OH *versus* good OH and poor OH *versus* good OH on periodontitis, with pooled odds ratios (ORs) of 2.04 [95% confidence interval (CI): 1.65–2.53] and 5.01 (95% CI: 3.40–7.39), respectively. Eleven studies examined oral care habits measured according to toothbrushing regularity and dental visit frequency; pooled ORs of 0.66 (95% CI: 0.47–0.94) and 0.68 (95% CI: 0.47–0.98) were obtained, respectively.

**Conclusions:**

Fair to poor OH increases the risk of periodontitis by two‐ to five‐fold. This risk can be reduced by regular toothbrushing and dental visits.

## Introduction

Periodontitis is the most common oral disease worldwide, with an age‐standardised prevalence of 11.2%^1^. It is a multifactorial disease^2^, with risk factors such as diabetes mellitus (DM), smoking and, most commonly, inadequate oral hygiene (OH)^3^. The accumulation of dental plaque and calculus is usually caused by improper toothbrushing techniques, failure to carry out interdental cleaning and irregular dental visits. This accumulation predictably results in gingival inflammation. Persistent gingivitis is a key risk predictor for the breakdown of periodontal attachment. Although poor OH is a well‐accepted and important risk factor for periodontitis, the magnitude of the association between OH and periodontitis has not yet been explored in a meta‐analysis. Therefore, we conducted a systematic review and meta‐analysis aiming to estimate the effects of OH on periodontitis, as measured by the Oral Hygiene Index (OHI), Plaque Index (PI) and plaque score (PSc). A second aim was to pool the magnitudes of association between oral care habits (regular toothbrushing, interdental cleaning and dental visits) and periodontitis.

## Methods

The Preferred Reporting Items for Systematic reviews and Meta‐Analyses (PRISMA) guidelines for conducting a meta‐analysis were followed^4^. The checklist is provided in *Appendix* S1 (PROSPERO registration number: CRD42015019036).

### Search strategy

Relevant studies were identified from Medline and Scopus databases, searched up to May 2016 using standardised methodological filters. Search strategies were mainly constructed based on the primary objective with three domains (i.e. periodontitis, OH and general aspects for observational studies), as follows: (‘periodontitis’ OR ‘periodontal’) AND (‘poor oral hygiene’ OR ‘plaque index’ OR ‘oral hygiene index’ OR ‘plaque score’) AND (‘relation’ OR ‘association’ OR ‘risk factor’). The search terms and strategies are described in *Table* S1.

### Inclusion criteria

Studies were screened based on titles and abstracts; if a decision could not be made based on this information, full papers were reviewed. Any type of observational study (e.g. cohort, case–control or cross‐sectional) published in English was included if it met the following criteria: (i) assessed associations between OH and periodontitis in either general or specific types of adult populations; (ii) had at least two outcome groups, namely periodontitis *versus* non‐periodontitis, or mild, moderate and severe periodontitis *versus* normal periodontium; (iii) assessed OH using standard tools, such as the OHI or Simplified Oral Hygiene Index (OHI‐S)^5^, PI^6^, plaque control record/PSc^7^ or a questionnaire including the frequency of brushing, interdental cleaning and dental visits; (iv) reported/possibly calculated the mean and standard deviation (SD) of OH scores among periodontitis groups or a contingency table between non‐periodontitis/periodontitis and OH groups. Studies were excluded if they had insufficient data for pooling after contacting the authors for additional data.

Two of three reviewers (A.L., S.R. and S.A.) independently evaluated the studies for eligibility, extracted the data and assessed the risk of bias. Any discrepancies between reviewers were discussed and resolved by consensus.

### Study factors

The primary study factor was OH, objectively measured using the OHI, PI or PSc. Secondary study factors were oral care habits, which were subjectively assessed using questionnaires assessing the frequency of toothbrushing, interdental cleaning and dental visits.

### Outcome

The outcome of interest was periodontitis, which was defined according to the original studies. The definition of periodontitis was based on periodontal probing depth, clinical attachment level or radiographs without a restricted periodontitis definition.

### Data extraction

Study characteristics, including study design (cohort, case–control or cross‐sectional), population type (general population or specific disease) and study location (community or hospital) were extracted. Subject characteristics (i.e. percentage of male subjects, smoking habits and the presence of DM) and clinical data (i.e. periodontitis definition and details of OH assessments) were also extracted.

### Risk of bias assessment

The quality of the studies was assessed using the modified Newcastle–Ottawa Quality Assessment Scale^8^ (*Appendix* S2), which considers three domains: the *representativeness* of the studied subjects; the *comparability* between groups; and the ascertainment of *outcome and exposure*. Each domain was graded by assigning stars if there was a low risk of bias. Individual studies were categorised, according to these stars, as having a low, moderate or high risk of bias if the percentage of stars was ≥75%, 50–74% and <50%, respectively.

### Statistical analysis

Data were pooled if there were at least two studies reporting the same outcomes and study factors. Data analysis was performed separately according to the type of OH data (i.e. categorical or continuous data), as described below.

For categorical data, the odds ratio (OR) of having periodontitis for fair OH *versus* good OH (OR_1_) and poor OH *versus* good OH (OR_2_), along with their 95% confidence intervals (95% CIs) were estimated for each study. For studies with two or more OH groups, a multivariate random‐effects meta‐analysis was applied for pooling ORs. This method considers within‐study variation using Riley's method^9^, ^10^. For studies in which OH was divided into more than two groups and ORs were reported without frequency data, the variance‐covariance was assumed to be zero.

For continuous data, the mean difference in OH scores between periodontitis and non‐periodontitis groups was estimated and pooled using a standardised mean difference (SMD). If logistic model correlation coefficients were reported instead of the mean and SD, the beta coefficients were then pooled using the pooling mean method.

Heterogeneity was assessed using Cochrane's *Q* test and the *I* ^2^ statistic. If heterogeneity was present (*Q* test <0.1 or *I* ^2^ ≥ 25%), a random‐effects model (DerSimonian and Laird)^11^ was used. Otherwise, a fixed‐effects model was applied using the inverse variance method.

Sources of heterogeneity were explored using a Galbraith plot to identify outlier studies. Covariables (i.e. population type, age, gender, smoking, DM, index use, periodontitis definition) were then fitted one‐by‐one into a meta‐regression model. If there was a suggested association, a sensitivity analysis excluding the outlier studies and/or a subgroup analysis was performed.

Finally, potential publication bias was explored using the Egger test and a funnel plot. If either of these indicated asymmetry, a contour‐enhanced funnel plot was constructed to identify the cause of asymmetry. All analyses were performed using STATA software version 14 (StataCorp, College Station, TX, USA). Two‐sided *P *<* *0.05 was considered statistically significant except for the heterogeneity test, in which *P *<* *0.10 was used.

### Grade of evidence

The system from the Grades of Recommendation, Assessment, Development and Evaluation Working Group (GRADE Working Group)^12^, ^13^ was used for grading the quality of evidence mainly based on the study design, risk of bias, indirectness of evidence, publication bias, heterogeneity and imprecision of results.

## Results

### Identifying studies

**Figure 1:**
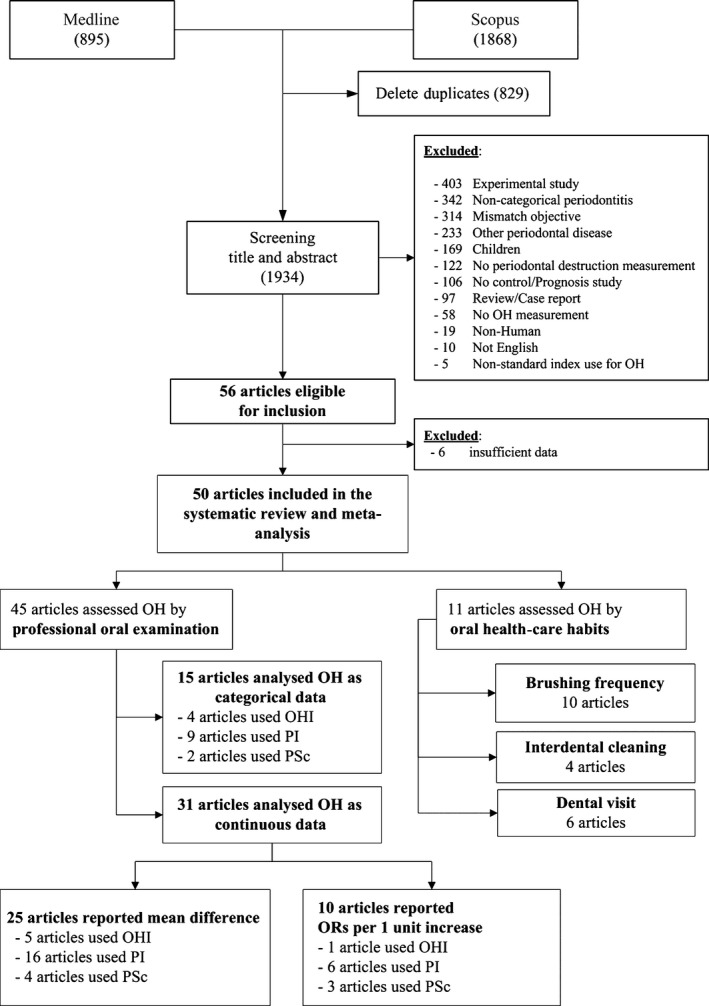
Flow chart of identification and selection of studies. OH, oral hygiene; OHI, Oral Hygiene Index; PI, Plaque Index; PSc, plaque score.

A total of 2,763 studies were identified from Medline and Scopus, and 1,934 studies remained after removing duplicates. Of these, 1,878 studies were ineligible for reasons described in *Figure* 1, leaving 56^14^, ^15^, ^16^, ^17^, ^18^, ^19^, ^20^, ^21^, ^22^, ^23^, ^24^, ^25^, ^26^, ^27^, ^28^, ^29^, ^30^, ^31^, ^32^, ^33^, ^34^, ^35^, ^36^, ^37^, ^38^, ^39^, ^40^, ^41^, ^42^, ^43^, ^44^, ^45^, ^46^, ^47^, ^48^, ^49^, ^50^, ^51^, ^52^, ^53^, ^54^, ^55^, ^56^, ^57^, ^58^, ^59^, ^60^, ^61^, ^62^, ^63^, ^64^, ^65^, ^66^, ^67^, ^68^, ^69^ that were eligible for review. Six studies^14^, ^47^, ^48^, ^51^, ^52^, ^57^ were excluded because of insufficient data after contacting the authors. Of the remaining 50 studies, 45^15^, ^16^, ^17^, ^18^, ^20^, ^21^, ^22^, ^23^, ^24^, ^25^, ^26^, ^27^, ^28^, ^29^, ^30^, ^31^, ^33^, ^35^, ^36^, ^37^, ^38^, ^39^, ^42^, ^43^, ^44^, ^45^, ^46^, ^49^, ^50^, ^53^, ^54^, ^55^, ^56^, ^58^, ^59^, ^60^, ^61^, ^62^, ^63^, ^64^, ^65^, ^66^, ^67^, ^68^, ^69^ objectively assessed OH using an oral examination. Of these 45 studies, 15^15^, ^17^, ^22^, ^26^, ^27^, ^28^, ^29^, ^31^, ^35^, ^36^, ^37^, ^38^, ^39^, ^46^, ^65^ analysed OH as categorical data, 31^16^, ^18^, ^20^, ^21^, ^22^, ^23^, ^24^, ^25^, ^30^, ^33^, ^42^, ^43^, ^44^, ^45^, ^49^, ^50^, ^53^, ^54^, ^55^, ^56^, ^58^, ^59^, ^60^, ^61^, ^62^, ^63^, ^64^, ^66^, ^67^, ^68^, ^69^ as continuous data and one^22^ as both. Eleven studies provided the association between periodontitis and oral care habits measured according to the frequency of brushing^29^, ^32^, ^33^, ^34^, ^36^, ^37^, ^40^, ^41^, ^44^, ^56^, interdental cleaning^29^, ^41^, ^44^, ^56^ and dental visits^19^, ^33^, ^34^, ^36^, ^40^, ^56^.

### Subject characteristics

The characteristics of the 50 included studies are described in *Table* 1. Most study designs were cross‐sectional, most studies investigated a general population and 34 were based in hospitals. The mean subject age ranged from 15 to 65 years. The percentages of male subjects, smokers and people with diabetes are also shown in *Table* 1. While the definition of periodontitis varied across the studies, most (92%) used periodontal probing depth and/or clinical attachment level.

**Table 1:** Characteristics of included studies

Authors	Study type	Study base	Population	OH measurement	Age	Male (%)	Smoking (%)	DM (%)	Periodontitis definition
Imaki^15^	Cross‐sectional	Community	General	PI	38.1	100	56.1	N/A	CPITN: 3–4
Norderyd^16^	Cross‐sectional	Community	General	PSc	48	48.7	20	4	Radiography: bone loss more than one‐third of root length
Wakai^17^	Cross‐sectional	Hospital	General	PI	51.1	82.1	34.4	N/A	CPITN: 3–4
Papapanou^18^	Case–control	Hospital	General	PSc	50.9	47.3	32.2	N/A	One site or more with PPD ≥5 mm AND CAL ≥3 mm
Hashim^19^	Cohort	Community	General	OHI, Dental visit	15	54.2	33.3	N/A	One site or more with ≥4 mm increase in CAL
Tezal^20^	Cross‐sectional	Community	General	PI	48.7	48.2	61.8	N/A	Mean CAL ≥2 mm
Hugoson^21^	Cross‐sectional	Community	General	PSc	65.0	52.7	42.9	N/A	Radiography: bone loss more than one‐third of root length
Do^22^	Cross‐sectional	Community	General	PI	40	40.3	28.9	N/A	Two or more sites with CAL ≥5 mm AND one site or more with PPD ≥4 mm
Meisel^23^	Cross‐sectional	Community	General	PSc	51.0	46.6	49.5	6	4th–5th quintiles of the percentage of sites with CAL >4 mm
Alpagot^24^	Cohort	Hospital	Patients with HIV	PI	34.1	57.9	N/A	0	One site or more with PPD ≥4 mm OR CAL ≥2 mm
Solis^25^	Cross‐sectional	Hospital	General	PI	37.4	35.3	23.5	0	Two or more sites with CAL ≥6 mm AND one site or more with PPD ≥5 mm
Wickholm^26^	Cross‐sectional	Community	General	PI	36.7	49.2	44.7	N/A	Three or more teeth with PPD ≥5 mm
Natto^27^	Cross‐sectional	Community	General	PI	36.4	64.9	70	N/A	≥10 sites with PPD ≥5 mm
Torrungruang^28^	Cross‐sectional	Community	General	PSc	60	74.4	14.3	15.8	Mean CAL >2.5 mm
de Macêdo^29^	Cross‐sectional	Community	General	PSc, Flossing, Brushing	N/A	33.8	31.4	N/A	Four or more teeth with PPD ≥4 mm AND CAL ≥3 mm at the same site
Khader^30^	Cross‐sectional	Hospital	General	PI	39.4	44.8	N/A	N/A	Khader's risk score
Vandana^31^	Cross‐sectional	Hospital	Dental fluorosis	OHI	25.36	68.6	N/A	0	CPITN: 3–4
Wang^32^	Cross‐sectional	Community	General	Brushing	N/A	45.7	27.7	0	Mean CAL ≥3 mm
Akhter^33^	Case–control	Hospital	General	PI, Brushing, Dental visit	38.5	50	45.7	N/A	Two or more sites with CAL ≥6 AND one site or more with PPD ≥5 mm
Kumar^34^	Cross‐sectional	Community	General	Brushing, Dental visit	33.9	100	N/A	N/A	CPITN: 3–4
Benguigui^35^	Cross‐sectional	Community	General	PI	58	54.9	19.2	6.7	CDC/AAP
Saxlin^36^	Cohort	Community	General	PI, Brushing, Dental visit	41.86	27	0	0	New teeth with PPD ≥4 mm
Bawadi^37^	Cross‐sectional	Hospital	General	PI, Brushing	36.4	49.4	20.3	17.9	Four or more teeth with PPD ≥4 mm AND CAL ≥3 mm at the same site
Carrilho Neto^38^	Cross‐sectional	Hospital	Inpatients	OHI	45.7	59.7	42.7	N/A	One site or more with PPD >4 mm
Mathur^39^	Cross‐sectional	Hospital	General	OHI	N/A	57.3	N/A	N/A	N/A
Teng^40^	Cross‐sectional	Hospital	Psychiatric inpatients	Brushing, Dental visit	41	62.5	42.5	N/A	CPITN: 3–4
Crocombe^41^	Cross‐sectional	Community	General	Brushing, Flossing	N/A	50	15	4.3	One site or more with CAL ≥4 mm
Mannem^42^	Cross‐sectional	Hospital	General	PI	52.5	44.1	34.2	N/A	Four or more teeth with PPD ≥4 mm AND CAL ≥3 mm at the same site
Raja^43^	Cross‐sectional	Hospital	General	PI	36.5	53.3	96.7	0	Four or more sites with CAL ≥4 mm
Vogt^44^	Cross‐sectional	Hospital	Pregnancy	PSc, Flossing, Brushing	27.2	0	15.87	0	Four or more teeth with PPD ≥4 mm AND CAL ≥4 mm at the same site
Fiyaz^45^	Case–control	Hospital	General	OHI	N/A	N/A	0	0	One site or more with PPD >4 mm OR CAL >1.5 mm
Palle^46^	Cross‐sectional	Hospital	CVD	OHI	57.2	84.1	32.3	52.2	Five or more sites with CAL ≥5 mm
Cakmak^49^	Case–control	Hospital	General	PI	38.3	49.1	0	0	One site or more with PPD ≥5 mm AND CAL ≥4 mm
Develioglu^50^	Case–control	Hospital	General	PI	46.7	N/A	0	33.3	≥30% sites with PPD ≥5 mm AND CAL ≥3 mm
Jacob^53^	Case–control	Hospital	General	PI	37.3	75.6	33.3	0	CDC/AAP
Kaur^54^	Case–control	Hospital	General	PI	N/A	66.7	25	0	N/A
Koseoglu^55^	Case–control	Hospital	General	PI	34.0	50	0	0	Four or more teeth with PPD ≥5 mm AND CAL ≥4 mm in each jaw
Kovačević^56^	Cross‐sectional	Hospital	General	OHI, Brushing, Flossing, Dental visit	38.9	77.2	31.7	N/A	CPITN: 3–4
Lavu^58^	Case–control	Hospital	General	OHI	33.6	50.4	0	0	CAL >1 mm at least 30% sites
Lutfioglu^59^	Case–control	Hospital	General	PI	33.1	53.3	51.1	0	One site or more with PPD ≥5 mm with radiographic evidence of bone loss
Meenawat^60^	Case–control	Hospital	General	PI	43.2	100	41.4	0	Four or more teeth with PPD >4 mm AND CAL >2 mm
Mesa^61^	Case–control	Hospital	General	PSc	46.3	40.3	46.8	N/A	Four or more teeth with PPD ≥4 mm AND CAL ≥3 mm at the same sites
Perayil^62^	Case–control	Hospital	General	OHI	43.1	43.3	0	0	Five or more teeth with PPD ≥5 mm AND CAL ≥3 mm
Pereira^63^	Case–control	Hospital	General	PSc	38.4	33.7	0	0	Four or more teeth with PPD ≥4 mm AND CAL ≥3 mm
Petrović^64^	Case–control	Hospital	General	PI	36.1	38.8	22.4	0	Thre or more quadrants with three or more sites with PPD ≥3 mm AND CAL ≥2 mm
Pranckeviciene^65^	Cross‐sectional	Hospital	Type I and type II DM	PI	43.86	N/A	25.9	100	One site or more with CAL >5 mm
Puri^66^	Case–control	Hospital	General	OHI	39.78	N/A	0	0	AAP 1999
Singh^67^	Case–control	Hospital	General	PI	43.5	52.5	0	0	One site or more with PPD ≥5 mm AND CAL ≥2 mm
Toyman^68^	Case–control	Hospital	General	PI	34.6	51.2	0	0	Six teeth or more with PPD ≥5 mm with radiographic evidence of bone loss
Varghese^69^	Case–control	Hospital	General	PI	N/A	65.3	0	0	≥30% sites with PPD ≥6 mm AND CAL ≥5 mm

CAL, clinical attachment level; CDC/AAP, periodontitis definition of the Centers for Disease Control and Prevention in collaboration with the American Academy of Periodontology; CPITN, the Community Periodontal Index of Treatment Needs; CVD, cardiovascular disease; DM, diabetes mellitus; HIV, human immunodeficiency virus; N/A, not available; OH, oral hygiene; OHI, oral hygiene index; PI, plaque index; PPD, periodontal pocket depth; PSc, plaque score.

### Risk of bias assessment

The results of the risk of bias assessments are described in *Table* S2. Most (72%) studies provided inadequate details for sample selection; hence, representativeness was unclear. For example, some authors did not mention their sampling methods or clearly describe their process for selecting cases and controls. Twenty‐seven (46%) studies were potentially biased because of improper statistical adjustments for confounding factors. Almost all studies measured periodontitis via an oral examination, which was objective and valid. However, 16 (32%) studies used partial‐mouth examination protocols, 16 (32%) studies diagnosed periodontitis without data regarding clinical attachment level and 25 (50%) studies did not provide details about intra/interexaminer agreement. The numbers of studies with low, moderate and high risks of bias were 23, 19 and 8, respectively.

### Oral hygiene

**Figure 2:**
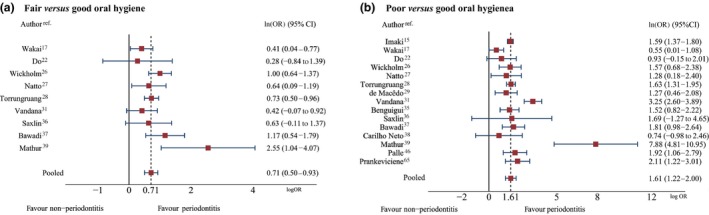
Pooling effects of fair oral hygiene (OH) *versus* good OH (a) and poor OH *versus* good OH (b) on periodontitis. 95% CI, 95% confidence interval; OR, odds ratio.

Of the 15 studies which reported OH as categorical data, six^15^, ^29^, ^35^, ^38^, ^46^, ^65^ categorised OH as good or poor, whereas nine^17^, ^22^, ^26^, ^27^, ^28^, ^31^, ^36^, ^37^, ^39^ categorised OH as good, fair or poor. The criteria for classifying OH are presented in *Table* S3. Pooled ln(ORs) determined using a multivariate meta‐analysis (*Figure* 2) were 0.71 (95% CI: 0.50–0.93) and 1.61 (95% CI: 1.22–2.00), which yielded pooled ORs of 2.04 (95% CI: 1.65–2.53) and 5.01 (95% CI: 3.40–7.39), respectively, for fair OH and poor OH. These results indicate that fair OH and poor OH increase the risk of periodontitis by approximately two‐ and five‐fold compared with good OH with an *I* ^2^ of 40% and 78%, respectively. The details of each individual study are shown in *Table* S4.

Population type appeared to be a large source of heterogeneity. Subgroup analyses in community‐based studies yielded lower heterogeneity levels [i.e. the *I* ^2^ values were 4% and 0% for fair and poor *versus* good OH, respectively, with corresponding pooled ORs of 2.23 (95% CI: 1.85–2.69) and 4.78 (95% CI: 4.10–5.58)]. In addition, a sensitivity analysis focussing on 11 studies^15^, ^17^, ^22^, ^26^, ^27^, ^28^, ^29^, ^35^, ^36^, ^37^, ^39^ of general populations decreased the degree of heterogeneity to 22% and 49% for fair OH *versus* good OH and poor OH *versus* good OH, with pooled ORs of 2.10 (95% CI: 1.76–2.49) and 4.21 (95% CI: 3.21–5.51), respectively. Moreover, the periodontitis definitions and index types used, as well as smoking behaviour, also contributed to heterogeneity (*Table* S5).

Among 31 studies that measured OH on a continuous scale, 25^18^, ^24^, ^25^, ^33^, ^42^, ^43^, ^44^, ^45^, ^49^, ^50^, ^53^, ^54^, ^55^, ^56^, ^58^, ^59^, ^60^, ^61^, ^62^, ^63^, ^64^, ^66^, ^67^, ^68^, ^69^ compared OH between periodontitis and non‐periodontitis groups using the mean scores. The SMDs were highly heterogeneous (*I* ^2^ = 95.6%), with a pooled SMD of 2.04 (95% CI: 1.59–2.50) (*Table* S6). From these findings, it could be interpreted that periodontitis subjects had a significantly higher OH score of 2.04 standardised units than did non‐periodontitis subjects.

**Figure 3:**
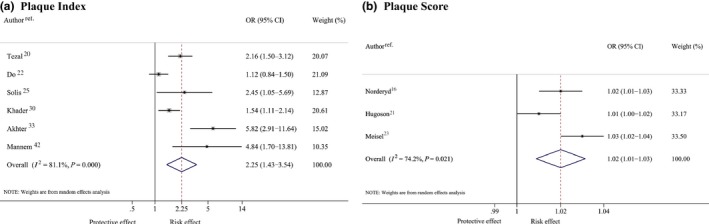
Pooling odds ratios (ORs) of plaque index (a) and plaque score (b) on periodontitis.

Six^20^, ^22^, ^25^, ^30^, ^33^, ^42^ and three^16^, ^21^, ^23^ studies reported the effects of PI and PSc on periodontitis as coefficients [i.e. ln(OR)] of logistic regression models. Pooling these corresponding effects yielded pooled ORs of 2.25 (95% CI: 1.43–3.54) and 1.02 (95% CI: 1.01–1.03), and high heterogeneity was found for both (*Figure* 3). These findings could be interpreted to indicate that each one‐unit increase in the measures of PI and PSc would increase the odds of having periodontitis by 2.25 and 1.02, respectively.

### Oral health‐care habits

**Figure 4:**
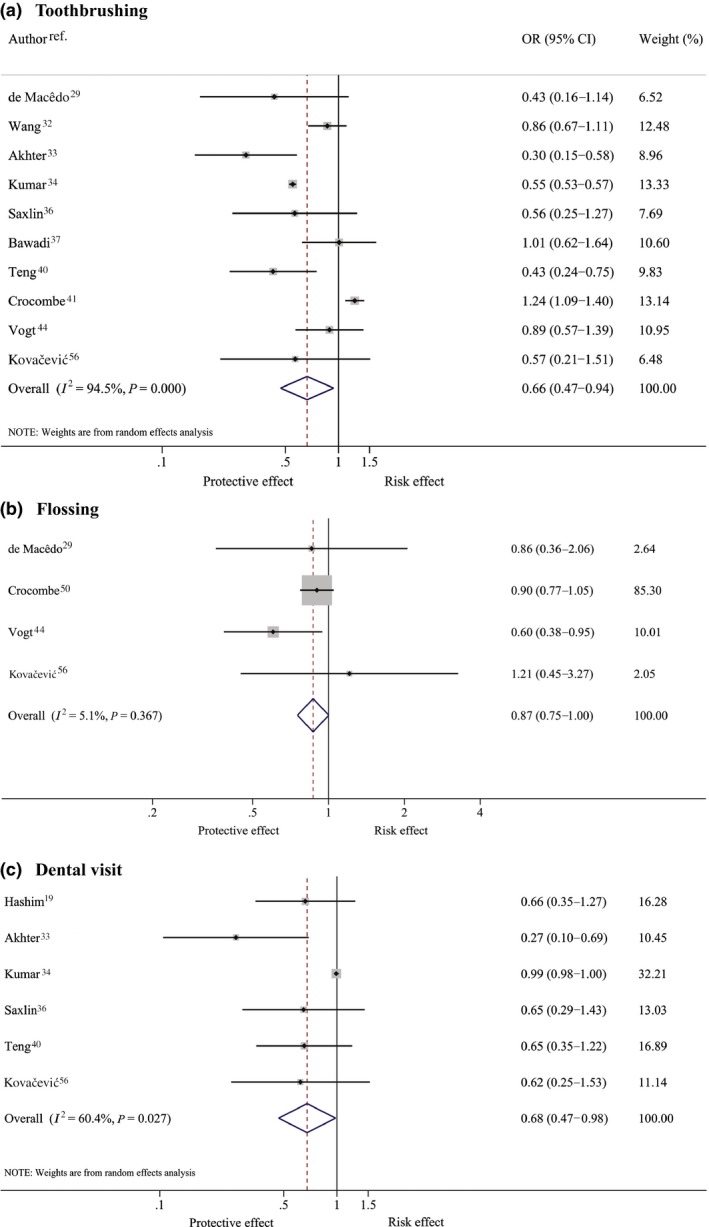
Pooling effect of oral care habits – toothbrushing (a), flossing (b) and dental visits (c) –on periodontitis.

Ten^29^, ^32^, ^33^, ^34^, ^36^, ^37^, ^40^, ^41^, ^44^, ^56^, four^29^, ^41^, ^44^, ^56^ and six^19^, ^33^, ^34^, ^36^, ^40^, ^56^ studies assessed the effects of brushing, dental floss and dental visits on periodontitis (*Table* S7). The pooled ORs (*Figure* 4) suggested that toothbrushing and dental visits were significantly associated with periodontitis, although the *I* ^2^ values showed high heterogeneity, at 94.5% and 60.4%, respectively. Subjects who brushed their teeth regularly had approximately 34% significantly lower odds of having periodontitis (pooled OR = 0.66; 95% CI: 0.47–0.94). Smoking, the definition of regular brushing and periodontitis were potential sources of heterogeneity (*Table* S8).

For dental visits, the sensitivity analysis was performed by considering four of six studies that had clearly defined a regular dental visit as at least one visit per year^19^, ^33^, ^36^, ^56^. This yielded a significant effect size of 0.56 (95% CI: 0.37–0.83) with an *I* ^2^ of 0%, indicating that subjects who regularly visited dentists at least once a year had a 44% lower risk of periodontitis than those who did not. The effects of interdental cleaning with dental floss on periodontitis showed little heterogeneity (*I* ^2^ = 5.1%), but the pooled OR was borderline significant (OR = 0.87; 95% CI: 0.75–1.00).

### Publication bias

Publication bias was assessed for all pooled estimates using funnel plots (*Figure* S1) and Egger tests (*Table* S9). The results suggested symmetry except for the mean differences in OH score, PSc and dental visits. Contour‐enhanced funnel plots were further constructed (*Figure* S2), and these indicated that the asymmetry of the funnels might be caused by both heterogeneity and publication bias.

### Quality of evidence

The scoring using the GRADE framework is shown in *Table* 2 and *Appendix* S3. Based on observational studies, all pooled estimates were graded as low quality^13^. For the effects of fair OH and poor OH on periodontitis, this was upgraded to moderate quality because of large effect sizes and strong dose–response relationships. The effects of brushing and dental visits were downgraded to very low quality caused by heterogeneity and publication bias, respectively.

**Table 2:** Overview of the meta‐analysis

Risk factor	No. of studies	Pooled OR (95% CI)	*I* ^2^ (%)	Quality of evidence^a^
**OH**				
Categorical data				
Fair OH *versus* Good OH	9	2.04 (1.65–2.53)	40	
Poor OH *versus* Good OH	15	5.01 (3.40–7.39)	78	
Continuous data				Moderate
PI: 1‐unit increase	6	2.25 (1.43–3.54)	81.1	
PSc: 1‐unit increase	3	1.02 (1.01–1.03)	74.2	
OH score	25	2.04 (1.59–2.50)^b^	95.6	
**Oral health‐care habits**				
Toothbrushing	10	0.66 (0.47–0.94)	94.5	Very low
Interdental cleaning	4	0.87 (0.75–1.00)	5.1	Low
Dental visits	6	0.68 (0.47–0.98)	60.4	Very low

OH, oral hygiene; PI, plaque index; PSc, plaque score.

a Quality of evidence: The Grades of Recommendation, Assessment, Development and Evaluation Working Group (GRADE Working Group).

b Pooled standard mean difference (SMD).

## Discussion

**Figure 5:**
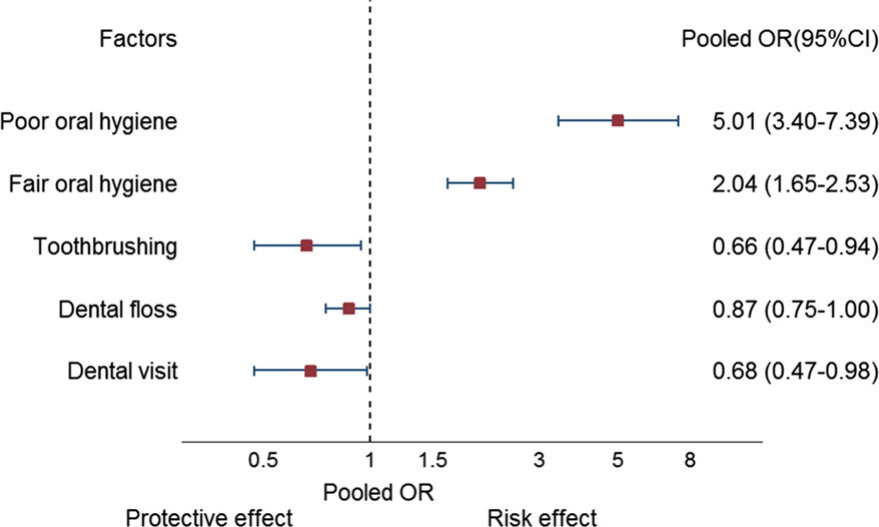
Summary of pooled effect of oral hygiene (OH) and oral care habits on periodontitis. OR, odds ratio.

We conducted a systematic review and meta‐analysis of the effects of OH on periodontitis. The results suggest a dose–response relationship between OH and periodontitis, with fair and poor OH significantly increasing the risk of having periodontitis by two‐ and five‐fold, respectively, compared with good OH. In contrast, regular toothbrushing and dentist visits could reduce periodontitis by 34% and 32%, respectively. These pooled OH effects and oral care habits are summarised in *Table* 2 and *Figure* 5.

The effect of OH on periodontitis was stronger than those of other risk factors, such as DM^70^ (OR = 2.6; 95% CI: 1.0–6.6), smoking^71^ (OR = 2.82; 95% CI: 2.36–3.39) or obesity^72^ (OR = 2.13; 95% CI: 1.40–3.26). Our results also showed protective effects of regular toothbrushing, which were consistent with the findings of a previous meta‐analysis^73^ that reported a significant risk for severe periodontitis caused by infrequent brushing (OR = 1.44; 95% CI: 1.21–1.71). However, our study could only identify a small effect of interdental cleaning with dental floss (i.e. a non‐significant reduction of 13% in the risk of periodontitis). This result was also consistent with a previous meta‐analysis^74^, which found little benefit from self‐performed flossing on plaque or periodontal parameters.

Although the use of OH assessments varied between the included studies, approximately half commonly used the PI with similar cut‐off points. OH was defined as *poor* for a PI of >2 or if the patient had a moderate accumulation of soft deposits visible by the naked eye; and OH was defined as *fair* for PI values ranging from 1 to 2 or if the patient had a film of plaque adhering to the tooth as detected by disclosing solution or probe.

To address concerns about the varying quality of the individual studies, a sensitivity analysis was also performed, including only studies with a low risk of bias^22^, ^26^, ^27^, ^28^, ^29^, ^35^, ^36^, ^37^, ^46^, ^65^. The results showed little difference compared with those of the main analysis, but heterogeneity was much lower.

Good OH and oral care habits should be encouraged and promoted in public health campaigns. Dentists and dental hygienists should regularly educate, motivate and assess patients’ perceptions for improving oral health behaviours. Additionally, dental nurses or assistants should encourage and provide general, useful information. Repeated and individually tailored OH instructions are key elements in achieving gingival health. Goal setting, self‐monitoring and planning are effective interventions for improving OH‐related behaviours in patients with periodontitis. Recognising the benefits of behaviour changes, their own susceptibility and the deleterious effects of periodontitis are important messages in periodontitis prevention^75^.

Patients should be able to access dental care regularly for professional cleaning together with tailoring and monitoring their OH^75^. They should also be taught how to perform plaque removal efficiently. Generally, mechanical plaque controlled by twice‐daily toothbrushing with a fluoride‐containing dentifrice is an accepted recommendation. The proper duration of toothbrushing is also mentioned as an important determinant of plaque removal; therefore, it should be stressed during toothbrushing instruction^76^. The current scientific data show that dental floss is not effective as a tool for removal of interdental plaque. It requires the user to be instructed about specific skills in order to be more effective. Interdental brushes have been shown to be the most effective method for removal of interdental plaque^77^; however, the selection of interdental aids must be at the clinician's discretion based on a patient's needs and dexterity and the characteristics of a patient's interdental spaces.

This study has some strengths. It includes studies of the effects of OH using both objective and subjective assessments. The magnitudes of the effects were pooled and reported. The results of subgroup analyses (i.e. population type, study base, periodontitis definition and smoking) were also explored. We used rigorous pooling methods (multivariate random‐effects meta‐analysis), which considered the variance‐covariance between the studies.

However, this study also has some limitations. Our pooled ORs were based on summary data of observational studies. Some data were reported without adjusting for potential confounders; thus, the pooled results might be prone to bias. Moreover, the definition of periodontitis varied among studies, which resulted in high heterogeneity, although the subgroup analyses did reduce this effect. Furthermore, the assessments of publication bias using funnel plots and Egger tests with the low numbers of included studies in some meta‐analyses may not be valid. Failure to detect asymmetry cannot rule out a reporting bias or vice versa.

In conclusion, poor OH increases the risk of periodontitis by approximately two‐ to five‐fold compared with good OH. Oral care habits, including regular brushing and dental visits, can decrease the risk of periodontitis and should thus be promoted as a public health intervention.

### Conflicts of interest

The authors declare no conflicts of interest. This study received no external funding, apart from the support of the authors’ institution.

## Supporting information


**Figure S1.** Funnel plots of publication bias assessment.

**Figure S2.** Contour‐enhanced funnel plots.

**Table S1.** Search terms and search strategy.
**Table S2.** Risk of bias assessment.
**Table S3.** Categorisation of OH level.
**Table S4.** Pooling effects of fair and poor *versus* good OH on periodontitis.
**Table S5.** Subgroup and sensitivity analysis according to sources of heterogeneity of fair and poor *versus* good OH.
**Table S6.** Pooling SMD of OH scores between periodontitis and non‐periodontitis.
**Table S7.** Pooled effect size of oral care habits on periodontitis.
**Table S8.** Sources of heterogeneity of tooth brushing meta‐analysis.
**Table S9.** Publication bias assessment by Egger test.

**Appendix S1.** PRISMA checklist.
**Appendix S2.** Modified Newcastle‐Ottawa Quality Assessment Scale.
**Appendix S3.** GRADE approach.
