# Supplementary figures and images for: The association between oral hygiene and periodontitis: a systematic review and meta‐analysis
Source: Int Dent J. 2017 Jun 23;67(6):332–43. doi: 10.1111/idj.12317 (PMC5724709; doi:10.1111/idj.12317)

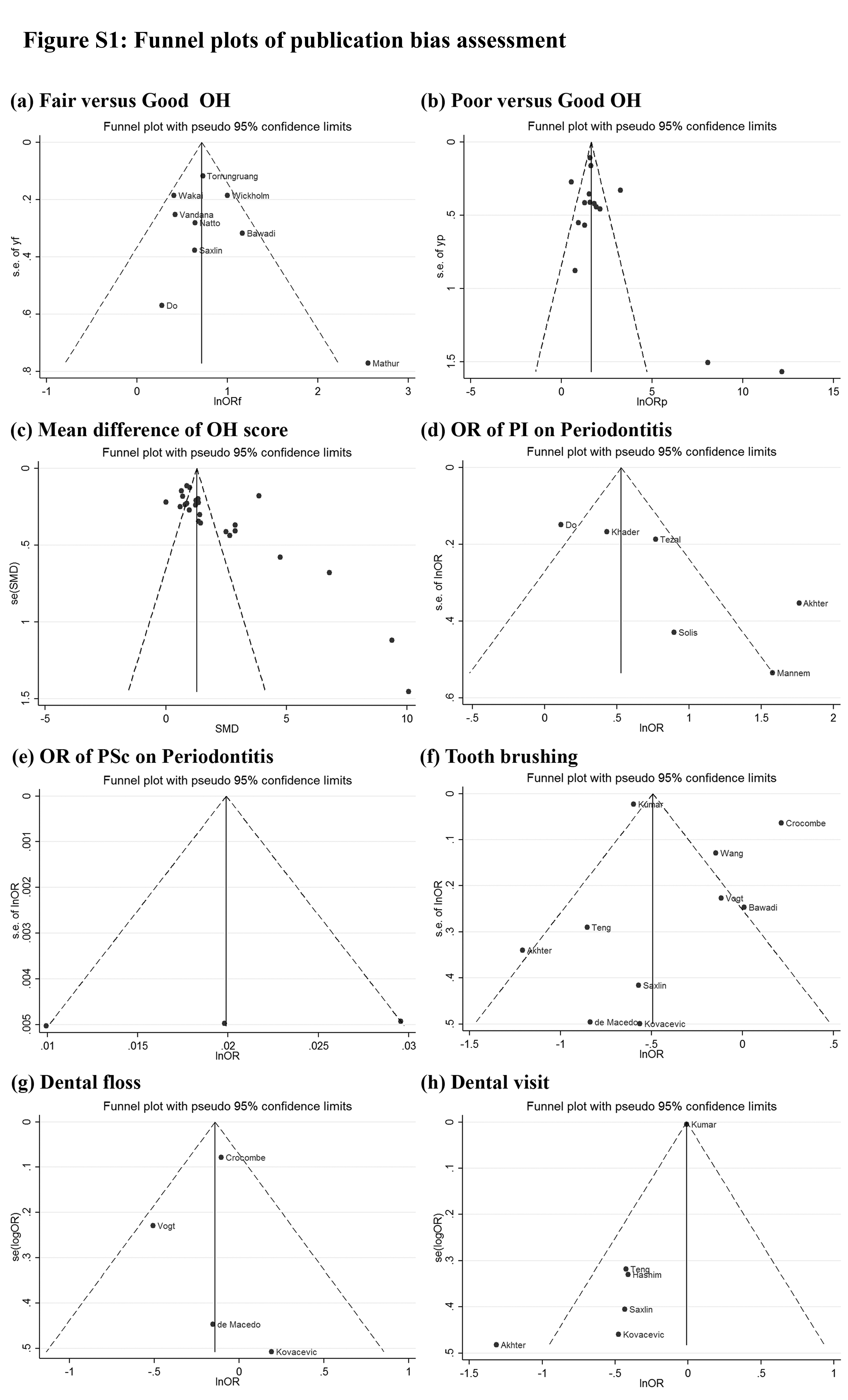

Supplement: Supplementary file 1 — Figure S1. Funnel plots of publication bias assessment. [file IDJ-67-332-s001.tif]

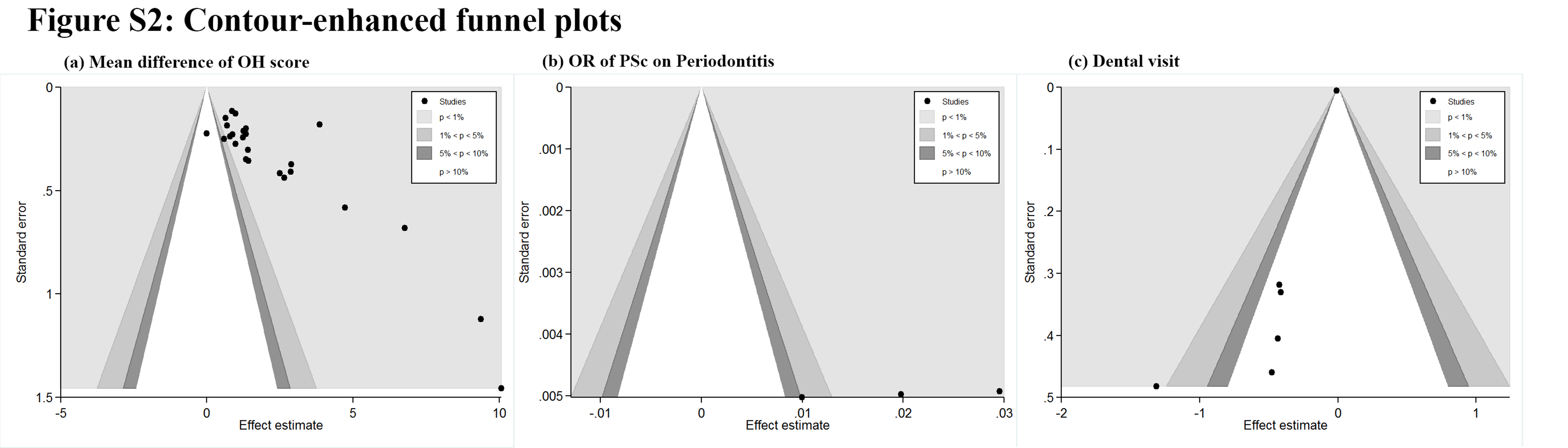

Supplement: Supplementary file 2 — Figure S2. Contour‐enhanced funnel plots. [file IDJ-67-332-s002.tif]
