# Supplementary material for: The association between oral hygiene and periodontitis: a systematic review and meta‐analysis
Source: Int Dent J. 2017 Jun 23;67(6):332–43. doi: 10.1111/idj.12317 (PMC5724709; doi:10.1111/idj.12317)
Supplement: Supplementary file 3 — Table S1. Search terms and search strategy. Table S2. Risk of bias assessment. Table S3. Categorisation of OH level. Table S4. Pooling effects of fair and poor versus good OH on periodontitis. Table S5. Subgroup and sensitivity analysis according to sources of heterogeneity of fair and poor versus good OH. Table S6. Pooling SMD of OH scores between periodontitis and non‐periodontitis. Table S7. Pooled effect size of oral care habits on periodontitis. Table S8. Sources of heterogeneity of tooth brushing meta‐analysis. Table S9. Publication bias assessment by Egger test. [file IDJ-67-332-s003.doc]

**Supplemental Tables**

**Supplemental legends**

Supplemental Tables

**Table S1:** Search terms and search strategy

**Table S2:** Risk of bias assessment

**Table S3:** Categorization of OH level

**Table S4:** Pooling effects of fair and poor versus good OH on periodontitis

**Table S5:** Subgroup and sensitivity analysis according to sources of heterogeneity of fair and poor versus good OH

**Table S6:** Pooling SMD of OH scores between periodontitis and non-periodontitis

**Table S7:** Pooled effect size of oral care habits on periodontitis

**Table S8:** Sources of heterogeneity of tooth brushing meta-analysis

**Table S9:** Publication bias assessment by Egger test

Supplemental information

**Supplemental information A:** PRISMA checklist

**Supplemental information B:** Modified Newcastle-Ottawa Quality Assessment Scale

**Supplemental information C:** GRADE approach

Supplemental Figures

**Figure S1:** Funnel plots of publication bias assessment

**Figure S2:** Contour-enhanced funnel plots

**Table S1** Search terms and search strategy

| **Item** | **Domains** | **Terms** |
| --- | --- | --- |
| **1** | **Periodontitis** | Periodontitis |
| **2** |  | Periodontal |
| **3** |  | Periodontitis [MesH]* |
| **4** |  | 1 OR 2 OR 3 |
| **5** | **Oral hygiene** | Poor oral hygiene |
| **6** |  | Plaque index |
| **7** |  | Dental plaque index [MeSH]* |
| **8** |  | Oral hygiene index |
| **9** |  | Oral hygiene index [MeSH]* |
| **10** |  | Plaque score |
| **11** |  | 5 OR 6 OR 7 OR 8 OR 9 OR 10 |
| **12** | **General** | Risk factor |
| **13** |  | Association |
| **14** |  | Relation |
| **15** |  | Correlation |
| **16** |  | 12 OR 13 OR 14 OR 15 |
| **17** |  | 4 AND 11 AND 16 |

* Options for MEDLINE

**Table S2 Risk of bias assessment**

| **Authors** | **Selection** | | | | | **Comparability** | | **Outcome/Exposure*** | | | | | | **Risk of bias** |
| --- | --- | --- | --- | --- | --- | --- | --- | --- | --- | --- | --- | --- | --- | --- |
|  | **S1** | **S2** | **S3** | **S4** | **S5** | **C1** | **C2** | **O1** | **O2** | **O3** | **O4** | **O5** | **O6** |  |
| Cohort study |  |  |  |  |  |  |  |  |  |  |  |  |  |  |
| Hashim^18^ | 1 | 1 | 1 | 0 | - | 1 | 1 | 1 | 0 | 1 | 0 | 1 | 1 | Low |
| Saxlin^35^ | 1 | 1 | 1 | 1 | - | 0 | 1 | 1 | 1 | 0 | 1 | 1 | 0.5 | Low |
| Case-control study |  |  |  |  |  |  |  |  |  |  |  |  |  |  |
| Akhter^32^ | 1 | 1 | 1 | 0 | 0.5 | 1 | 1 | 1 | 1 | 0 | - | - | - | Low |
| Fiyaz^44^ | 1 | 1 | 0 | 0 | 0 | 0 | 0 | 1 | 1 | 0 | - | - | - | High |
| Papapanou^17^ | 1 | 1 | 1 | 0 | 0.5 | 1 | 1 | 1 | 1 | 0 | - | - | - | Low |
| Cakmak^48^ | 1 | 1 | 0 | 0 | 0.5 | 1 | 1 | 1 | 1 | 0 | - | - | - | Mod |
| Develioglu^49^ | 1 | 0 | 0 | 0 | 0 | 1 | 0 | 1 | 1 | 0 | - | - | - | High |
| Jacob^52^ | 1 | 1 | 1 | 0 | 0.5 | 1 | 0 | 1 | 1 | 0 | - | - | - | Mod |
| Kaur^53^ | 0 | 0 | 0 | 0 | 0 | 1 | 0 | 1 | 1 | 0 | - | - | - | High |
| Koseoglu^54^ | 1 | 1 | 0 | 0 | 0.5 | 1 | 0 | 1 | 1 | 0 | - | - | - | Mod |
| Lavu^57^ | 1 | 1 | 1 | 0 | 0.5 | 1 | 1 | 1 | 1 | 0 | - | - | - | Low |
| Lutfioglu^58^ | 0 | 1 | 0 | 0 | 0.5 | 1 | 0 | 1 | 1 | 0 | - | - | - | High |
| Meenawat^59^ | 1 | 1 | 0 | 0 | 0 | 0 | 0 | 1 | 1 | 0 | - | - | - | High |
| Mesa^60^ | 1 | 1 | 1 | 1 | 1 | 1 | 1 | 1 | 1 | 0 | - | - | - | Low |
| Perayil^61^ | 1 | 1 | 1 | 0 | 1 | 1 | 0 | 1 | 1 | 0 | - | - | - | Mod |
| Pereira^62^ | 1 | 1 | 0 | 0 | 1 | 1 | 0 | 1 | 1 | 0 | - | - | - | Mod |
| Petrović^63^ | 1 | 0 | 1 | 0 | 0 | 1 | 0 | 1 | 1 | 0 | - | - | - | Mod |
| Puri^65^ | 1 | 1 | 0 | 0 | 0 | 1 | 0 | 1 | 1 | 0 | - | - | - | Mod |
| Singh^66^ | 1 | 1 | 0 | 0 | 0 | 0 | 1 | 1 | 1 | 0 | - | - | - | Mod |
| Toyman^67^ | 0 | 1 | 0 | 0 | 0.5 | 1 | 0 | 1 | 1 | 0 | - | - | - | High |
| Varghese^68^ | 1 | 1 | 0 | 0 | 0 | 1 | 0 | 1 | 1 | 0 | - | - | - | Mod |
| Cross-sectional study |  |  |  |  |  |  |  |  |  |  |  |  |  |  |
| Alpagot^23^ | 0 | 1 | - | - | - | 0 | 0 | 1 | 0 | 1 | 1 | - | - | Mod |
| Bawadi^36^ | 1 | 1 | - | - | - | 1 | 1 | 1 | 1 | 1 | 1 | - | - | Low |
| Benguigui^34^ | 1 | 1 | - | - | - | 0 | 0 | 1 | 1 | 1 | 1 | - | - | Low |
| Carrilho Neto^37^ | 0 | 1 | - | - | - | 1 | 1 | 1 | 1 | 0 | 0 | - | - | Mod |
| Crocombe^40^ | 1 | 1 | - | - | - | 1 | 1 | 1 | 1 | 1 | 0 | - | - | Low |
| Do^21^ | 1 | 1 | - | - | - | 1 | 1 | 1 | 0 | 1 | 1 | - | - | Low |
| Hugoson^20^ | 1 | 1 | - | - | - | 1 | 1 | 0 | 0 | 0 | 1 | - | - | Mod |
| Imaki^14^ | 0 | 1 | - | - | - | 1 | 1 | 1 | 0 | 0 | 0 | - | - | Mod |
| Khader^29^ | 0 | 1 | - | - | - | 1 | 1 | 1 | 1 | 1 | 1 | - | - | Low |
| Kovačević^55^ | 1 | 1 | - | - | - | 0 | 0 | 1 | 1 | 0 | 0 |  |  | Mod |
| Kumar^33^ | 1 | 1 | - | - | - | 0 | 1 | 1 | 0 | 0 | 1 | - | - | Mod |
| de Macedo^28^ | 0 | 1 | - | - | - | 1 | 1 | 1 | 1 | 1 | 1 | - | - | Low |
| Mannem^41^ | 0 | 1 | - | - | - | 1 | 1 | 1 | 1 | 1 | 1 | - | - | Low |
| Mathur^38^ | 0 | 1 | - | - | - | 0 | 0 | 1 | 1 | 0 | 0 | - | - | High |
| Meisel^22^ | 1 | 1 | - | - | - | 1 | 1 | 1 | 0 | 1 | 0 | - | - | Low |

**Table S2** Risk of bias assessment *(continued)*

| **Authors** | **Selection** | | | | | **Comparability** | | **Outcome/Exposure*** | | | | | | **Risk of bias** |
| --- | --- | --- | --- | --- | --- | --- | --- | --- | --- | --- | --- | --- | --- | --- |
|  | **S1** | **S2** | **S3** | **S4** | **S5** | **C1** | **C2** | **O1** | **O2** | **O3** | **O4** | **O5** | **O6** |  |
| Natto^26^ | 0 | 1 | - | - | - | 1 | 1 | 1 | 1 | 0 | 1 | - | - | Low |
| Norderyd^15^ | 1 | 1 | - | - | - | 1 | 1 | 0 | 0 | 0 | 0 | - | - | Mod |
| Palle^45^ | 0 | 1 | - | - | - | 1 | 1 | 1 | 1 | 1 | 0 | - | - | Low |
| Pranckeviciene^64^ | 0 | 1 | - | - | - | 0 | 1 | 1 | 1 | 1 | 1 | - | - | Low |
| Raja^42^ | 0 | 1 | - | - | - | 1 | 1 | 1 | 1 | 1 | 1 | - | - | Low |
| Solis^24^ | 0 | 1 | - | - | - | 1 | 1 | 1 | 1 | 1 | 1 | - | - | Low |
| Teng^39^ | 0 | 1 | - | - | - | 1 | 1 | 1 | 0 | 0 | 0 | - | - | Mod |
| Tezal^19^ | 1 | 1 | - | - | - | 1 | 1 | 1 | 1 | 1 | 1 | - | - | Low |
| Torrungruang^27^ | 0 | 1 | - | - | - | 1 | 1 | 1 | 0 | 1 | 1 | - | - | Low |
| Vandana^30^ | 0 | 1 | - | - | - | 0 | 0 | 1 | 0 | 0 | 0 | - | - | High |
| Vogt^43^ | 0 | 1 | - | - | - | 0 | 0 | 1 | 1 | 1 | 0 | - | - | Mod |
| Wakai^16^ | 0 | 1 | - | - | - | 1 | 1 | 1 | 0 | 0 | 0 | - | - | Mod |
| Wang^31^ | 1 | 1 | - | - | - | 1 | 1 | 1 | 0 | 1 | 1 | - | - | Low |
| Wickholm^25^ | 1 | 1 | - | - | - | 1 | 1 | 1 | 1 | 0 | 1 | - | - | Low |

***Outcome**: Cohort and Cross-sectional study || **Exposure**: Case-control study

**Table S3** Categorization of OH level

| **Authors** | **Index** | **Good OH** | **Fair OH** | **Poor OH** |
| --- | --- | --- | --- | --- |
| Mathur^38^ | OHI | < 1 | 1-1.99 | ≥ 2 |
| Vandana^30^ | OHI | < 1 | 1-1.99 | ≥ 2 |
| Carilho Neto^37^ | OHI | < 3 | - | ≥ 3 |
| Palle^45^ | OHI | < 2 | - | ≥ 2 |
| Bawadi^36^ | PI | < 1 | 1-1.99 | ≥ 2 |
| Do^21^ | PI | < 1 | 1-1.99 | ≥ 2 |
| Saxlin^35^ | PI | < 1 | 1-1.99 | ≥ 2 |
| Wakai^16^ | PI | < 1 | 1-1.99 | ≥ 2 |
| Wickholm^25^ | PI | < 1 | 1-1.99 | ≥ 2 |
| Benguigui^34^ | PI | Mean | - | >= Mean |
| Imaki^14^ | PI | ≤ 1 | - | > 1 |
| Pranckeviciene^64^ | PI | < 1 | - | ≥ 1 |
| Natto^26^ | PI | 0-0.69 | 0.7 -1.30 | ≥ 1.31 |
| de Macedo^28^ | Psc | 0-64 | - | ≥ 65 |
| Torrungruang^27^ | Psc | 0-39 | 40-79 | ≥ 80 |
|  |  |  |  |  |

**Table S4** Pooling effects of fair and poor versus good OH on periodontitis

| **Authors** | **Index** | **Periodontitis (N)** | | |  | **Non-Periodontitis (N)** | | |  | **OR_1_*** | **95% CI** | **I^2^** | **OR_2_**** | **95% CI** | **I^2^** |
| --- | --- | --- | --- | --- | --- | --- | --- | --- | --- | --- | --- | --- | --- | --- | --- |
|  |  | **Good OH** | **Fair OH** | **Poor OH** |  | **Good OH** | **Fair OH** | **Poor OH** |  |  |  |  |  |  |  |
| Vandana^30^ | OHI | 21 | 156 | 100 |  | 124 | 605 | 23 |  | 1.52 | 0.93, 2.50 |  | 25.67 | 13.44, 49.06 |  |
| Carilho Neto^37^ | OHI |  |  |  |  |  |  |  |  |  |  |  | 2.10 | 0.37, 11.76 |  |
| Mathur^38^ | OHI | 2 | 60 | 178 |  | 18 | 42 | 0 |  | 12.86 | 2.83, 58.38 |  | 2641.80^#^ | 122.16, 57129.99 |  |
| Palle^45^ | OHI |  |  |  |  |  |  |  |  |  |  |  | 6.85 | 2.87, 16.35 |  |
| **Pooled** |  |  |  |  |  |  |  |  |  | **6.16** | **0.85, 44.90** | **81%** | **7.64** | **2.14, 27.36** | **71%** |
| Imaki^14^ | PI | 186 |  | 553 |  | 542 |  | 330 |  |  |  |  | 4.88 | 3.94, 6.06 |  |
| Wakai^16^ | PI | 144 | 214 | 68 |  | 88 | 87 | 24 |  | 1.50 | 1.04, 2.16 |  | 1.73 | 1.01, 2.96 |  |
| Do^21^ | PI | 4 | 36 | 103 |  | 24 | 164 | 244 |  | 1.32 | 0.43, 4.03 |  | 2.53 | 0.86, 7.48 |  |
| Wickholm^25^ | PI |  |  |  |  |  |  |  |  | 2.72 | 1.89, 3.92 |  | 4.81 | 1.99, 10.80 |  |
| Natto^26^ | PI |  |  |  |  |  |  |  |  | 1.90 | 1.09, 3.30 |  | 3.60 | 1.20, 11.00 |  |
| Benguigui^34^ | PI | 87 |  | 114 |  | 42 |  | 12 |  |  |  |  | 4.59 | 2.28, 9.23 |  |
| Saxlin^35^ | PI | 55 | 44 | 4 |  | 33 | 14 | 0 |  | 1.89 | 0.90, 3.95 |  | 5.43^#^ | 0.28, 104.11 |  |
| Bawadi^36^ | PI | 15 | 69 | 21 |  | 83 | 119 | 19 |  | 3.21 | 1.72, 5.99 |  | 6.12 | 2.67, 14.01 |  |
| Pranckeviciene^64^ | PI | 98 |  | 122 |  | 40 |  | 6 |  |  |  |  | 8.30 | 3.38, 20.38 |  |
| **Pooled** |  |  |  |  |  |  |  |  |  | **2.26** | **1.75, 2.92** | **36%** | **4.15** | **3.00, 5.72** | **50%** |
| Torrungruang^27^ | PSc | 218 | 778 | 398 |  | 198 | 342 | 71 |  | 2.07 | 1.64, 2.60 |  | 5.09 | 3.71, 6.99 |  |
| de Macedo^28^ | PSc | 9 |  | 33 |  | 64 |  | 66 |  |  |  |  | 3.56 | 1.58, 8.02 |  |
| **OVERALL POOLED** | |  |  |  |  |  |  |  |  | **2.04** | **1.65, 2.53** | **40%** | **5.01** | **3.40, 7.39** | **78%** |

** Fair versus Good OH, ** Poor versus Good OH*

*^#^ calculated with continuity correction*

**Table S5** Subgroup and sensitivity analysis according to sources of heterogeneity of fair and poor versus good oral hygiene

| **Factors** | **Fair Vs Good OH** | | | **Poor vs Good OH** | | |
| --- | --- | --- | --- | --- | --- | --- |
|  | Number of studies | OR_1_ (95% CI) | I^2^ | Number of studies | OR_2_ (95% CI) | I^2^ |
| **Overall** | **9** | **2.04 (1.65, 2.53)** | **40%** | **15** | **5.01 (3.40, 7.39)** | **78%** |
| **Subgroup analysis**  *Type of index*  OHI  PI  PSc* | 2  6  1 | 6.16 (0.85, 44.90)  2.26 (1.75, 2.92)  - | 81%  36%  - | 4  9  2 | 7.64 (2.14, 27.36)  4.15 (3.00, 5.72)  - | 71%  50%  - |
| *Study-based*  Community  Hospital | 6  3 | 2.23 (1.85, 2.69)  2.43 (0.72, 8.17) | 4%  90% | 9  6 | 4.78 (4.10, 5.58)  6.06 (2.08, 17.68) | 0%  84% |
| *Periodontitis definition***  Using PD only  Using CAL or x-ray only*  Using PD with CAL | 5  1  2 | 1.86 (1.40, 2.46)  -  2.45 (1.58, 3.79) | 43%  -  0% | 7  3  4 | 4.86 (2.12, 11.18)  -  4.42 (2.98, 6.55) | 89%  -  0% |
| *Smoking***  < 25%  ≥ 25% | 3  4 | 2.15 (1.59, 2.91)  2.19 (1.64, 2.94) | 1%  40% | 4  9 | 5.05 (3.84, 6.62)  4.05 (2.89, 5.67) | 0%  53% |
| **Sensitivity analysis**  Only studies focused on  *general population* | 8 | 2.10 (1.76, 2.49) | 22% | 11 | 4.21 (3.21, 5.51) | 49% |

** Insufficient number of studies for pooling OR via mvmeta method*

*** Missing data: periodontitis definition (1 study), smoking (2 studies)*

**Table S6** Pooling SMD of OH score between periodontitis and non-periodontitis

| **Authors** | **Periodontitis** | | | **Non-Periodontitis** | | | **SMD** | **95% CI** |
| --- | --- | --- | --- | --- | --- | --- | --- | --- |
|  | **N** | **Mean** | **SD** | **N** | **Mean** | **SD** |  |  |
| **Oral hygiene index:** |  |  |  |  |  |  |  |  |
| Fiyaz^44^ | 30 | 2.38 | 0.81 | 30 | 0.53 | 0.41 | 2.88 | 2.15, 3.61 |
| Perayil^61^ | 30 | 3.89 | 0.68 | 30 | 0.53 | 0.17 | 6.78 | 5.45, 8.11 |
| Kovacevic^55^ | 73 | 1.18 | 0.64 | 28 | 1.18 | 0.64 | 0.00 | -0.44, 0.44 |
| Puri^65^ | 20 | 3.70 | 0.86 | 20 | 2.75 | 0.37 | 1.44 | 0.74, 2.13 |
| Lavu^57^ | 177 | 2.77 | 0.83 | 176 | 0.40 | 0.25 | 3.87 | 3.51, 4.22 |
| ***Pooled (OHI)**** | ***I^2^ = 98.9*** | |  |  |  |  | ***1.71*** | ***0.65, 2.78*** |
| **Plaque index:** |  |  |  |  |  |  |  |  |
| Alpagot^23^ | 111 | 1.79 | 0.74 | 41 | 0.88 | 0.47 | 1.34 | 0.95, 1.73 |
| Solis^24^ | 44 | 1.32 | 0.79 | 105 | 0.87 | 0.57 | 0.70 | 0.34, 1.06 |
| Akhter^32^ | 140 | 1.80 | 0.70 | 140 | 1.20 | 0.50 | 0.99 | 0.74, 1.23 |
| Mannem^41^ | 77 | 1.19 | 0.44 | 34 | 0.65 | 0.29 | 1.35 | 0.91, 1.79 |
| Raja^42^ | 30 | 1.60 | 0.33 | 30 | 1.31 | 0.26 | 0.98 | 0.44, 1.51 |
| Cakmak^48^ | 80 | 1.78 | 0.59 | 40 | 1.05 | 0.57 | 1.25 | 0.84, 1.66 |
| Develioglu^49^ | 32 | 2.23 | 0.52 | 16 | 0.19 | 0.10 | 4.74 | 3.60, 5.88 |
| Jacob^52^ | 30 | 1.35 | 0.39 | 15 | 0.48 | 0.24 | 2.50 | 1.68, 3.31 |
| Kaur^53^ | 20 | 1.69 | 0.35 | 20 | 0.75 | 0.36 | 2.65 | 1.79, 3.51 |
| Petrovic^63^ | 36 | 1.20 | 0.78 | 31 | 0.74 | 0.77 | 0.59 | 0.10, 1.08 |
| Singh^66^ | 20 | 2.48 | 0.27 | 20 | 0.57 | 0.10 | 9.38 | 7.18, 11.58 |
| Koseoglu^54^ | 20 | 1.52 | 0.30 | 40 | 0.93 | 0.47 | 1.41 | 0.82, 2.01 |
| Lutfioglu^58^ | 32 | 2.29 | 0.97 | 60 | 1.26 | 1.27 | 0.88 | 0.43, 1.32 |
| Meenawat^59^ | 24 | 2.49 | 0.23 | 5 | 0.33 | 0.08 | 10.07 | 7.22, 12.92 |
| Toyman^67^ | 21 | 0.89 | 0.90 | 20 | 0.02 | 0.06 | 1.35 | 0.67, 2.03 |
| Varghese^68^ | 25 | 1.29 | 0.41 | 25 | 0.29 | 0.27 | 2.88 | 2.08, 3.68 |
| ***Pooled (PI)**** | ***I^2^ = 98.3*** | |  |  |  |  | ***0.97*** | ***0.61, 1.32*** |
| **Plaque score:** |  |  |  |  |  |  |  |  |
| Papapanou^17^ | 131 | 51.20 | 24.50 | 74 | 35.00 | 25.60 | 0.65 | 0.36, 0.94 |
| Vogt^43^ | 157 | 73.80 | 14.20 | 177 | 59.60 | 17.90 | 0.87 | 0.65, 1.10 |
| Pereira^62^ | 31 | 79.00 | 17.90 | 58 | 49.20 | 26.80 | 1.24 | 0.76, 1.71 |
| Mesa^60^ | 41 | 74.47 | 34.79 | 36 | 46.32 | 35.36 | 0.80 | 0.34, 1.27 |
| ***Pooled (PSc)**** | ***I^2^ = 74.2*** | |  |  |  |  | ***20.44*** | ***12.85, 28.04*** |
| ***Overall Pooled*** | ***I^2^ = 95.6*** | |  |  |  |  | ***2.04*** | ***1.59, 2.50*** |

** Non-standardized mean difference pooling*

**Table S7** Pooled effect size of oral care habit on periodontitis

| **Author** | **Periodontitis (N)** | |  | **Non-Periodontitis (N)** | |  | **OR** | **95% CI** | **I^2^** |
| --- | --- | --- | --- | --- | --- | --- | --- | --- | --- |
|  | **Good Habits** | **Bad habits** |  | **Good Habits** | **Bad habits** |  |  |  |  |
| **Brushing** |  |  |  |  |  |  |  |  |  |
| de Macedo^28^ | 34 | 8 |  | 118 | 12 |  | 0.43 | 0.16, 1.14 |  |
| Wang^31^ |  |  |  |  |  |  | 0.86 | 0.67, 1.11 |  |
| Akhter^32^ | 14 | 126 |  | 38 | 102 |  | 0.30 | 0.15, 0.58 |  |
| Kumar^33^ |  |  |  |  |  |  | 0.55 | 0.53, 0.57 |  |
| Saxlin^35^ | 67 | 33 |  | 36 | 10 |  | 0.56 | 0.25, 1.27 |  |
| Bawadi^36^ | 69 | 36 |  | 154 | 81 |  | 1.01 | 0.62, 1.64 |  |
| Teng^39^ |  |  |  |  |  |  | 0.43 | 0.24, 0.75 |  |
| Crocombe^40^ | 1356 | 788 |  | 1179 | 847 |  | 1.24 | 1.09, 1.40 |  |
| Vogt^43^ | 56 | 101 |  | 68 | 109 |  | 0.89 | 0.57, 1.39 |  |
| Kovacevic^55^ | 46 | 27 |  | 21 | 7 |  | 0.57 | 0.21, 1.51 |  |
| **Pooled** |  |  |  |  |  |  | **0.66** | **0.47, 0.94** | **94.5%** |
| **Floss** |  |  |  |  |  |  |  |  |  |
| de Macedo^28^ | 8 | 34 |  | 28 | 102 |  | 0.86 | 0.36, 2.06 |  |
| Crocombe^40^ |  |  |  |  |  |  | 0.90 | 0.77, 1.05 |  |
| Vogt^43^ | 49 | 108 |  | 76 | 101 |  | 0.60 | 0.38, 0.95 |  |
| Kovacevic^55^ | 21 | 52 |  | 7 | 21 |  | 1.21 | 0.45, 3.27 |  |
| **Pooled** |  |  |  |  |  |  | **0.87** | **0.75, 1.00** | **5.1%** |
| **Dental visit** |  |  |  |  |  |  |  |  |  |
| Hashim^18^ |  |  |  |  |  |  | 0.66 | 0.35, 1.27 |  |
| Akhter^32^ | 6 | 134 |  | 20 | 120 |  | 0.27 | 0.10, 0.69 |  |
| Kumar^33^ |  |  |  |  |  |  | 0.99 | 0.98, 1.00 |  |
| Saxlin^35^ | 68 | 33 |  | 35 | 11 |  | 0.65 | 0.29, 1.43 |  |
| Teng^39^ |  |  |  |  |  |  | 0.65 | 0.35, 1.22 |  |
| Kovacevic^55^ | 38 | 34 |  | 18 | 10 |  | 0.62 | 0.25, 1.53 |  |
| **Pooled** |  |  |  |  |  |  | **0.68** | **0.47, 0.98** | **60.4%** |

**Table S8** Sources of heterogeneity of tooth brushing meta-analysis

| **Factors** | **Number**  **of studies** | **OR (95% CI)** | **I^2^** |
| --- | --- | --- | --- |
| Overall | 10 | 0.66 (0.47, 0.94) | 94.5% |
| Smoking*  < 25%  ≥ 25% | 4  5 | 1.03 (0.78, 1.35)  0.51 (0.31, 0.83) | 47%  69% |
| Definition of regular brushing  Once a day  Twice a day | 3  7 | 0.57 (0.35, 0.94)  0.75 (0.54, 1.05) | 78%  77% |
| Periodontitis definition  Using PD only  Using CAL or x-ray only  Using PD combined with CAL | 4  3  3 | 0.55 (0.53, 0.57)  0.94 (0.64, 1.39)  0.67 (0.35, 1.29) | 0%  80%  79% |

** Missing data of smoking: 1 study*

**Table S9** Publication bias assessment by Egger test

| **Pooling** | **N**  **(Study)** | **Coefficient** | **Std. error** | **P-value** |
| --- | --- | --- | --- | --- |
| **OH: Continuous data** | | | | |
| Mean difference | 25 | 6.00 | 1.75 | 0.002* |
| OR: Plaque index | 6 | 4.08 | 1.52 | 0.055 |
| OR: Plaque score | 3 | -201.87 | 0.57 | 0.002* |
| **OH: Categorical data** | | | | |
| OR: Poor Vs Good OH | 15 | 0.17 | 0.78 | 0.827 |
| OR: Fair Vs Good OH | 9 | 0.68 | 1.01 | 0.523 |
| **Oral health care habit** | | | | |
| Brushing | 10 | 1.30 | 1.71 | 0.468 |
| Floss | 4 | -0.32 | 0.96 | 0.771 |
| Dental visit | 6 | -1.48 | 0.32 | 0.010* |

** Significance suggested asymmetry pattern*
