# Supplementary material for: The association between oral hygiene and periodontitis: a systematic review and meta‐analysis
Source: Int Dent J. 2017 Jun 23;67(6):332–43. doi: 10.1111/idj.12317 (PMC5724709; doi:10.1111/idj.12317)
Supplement: Supplementary file 4 — Appendix S1. PRISMA checklist. Appendix S2. Modified Newcastle‐Ottawa Quality Assessment Scale. Appendix S3. GRADE approach. [file IDJ-67-332-s004.doc]

# The GRADE Approach

| **Pooling** | **Study design (No. of studies)** | **Risk of bias^1^** | **Inconsistency^2^** | **Indirectness** | **Imprecision** | **Other considerations (+/-)** | **Quality of Evidence** |
| --- | --- | --- | --- | --- | --- | --- | --- |
| **Fair and poor versus**  **good OH** | Observational (15):  - Cohort (1)  - Cross-sectional (14) | Not serious | Not serious | Not serious | Not serious | (+) Large effect^3^  (+) Dose-response  gradient^4^ | **(+)(+)(+)( )**  MODERATE |
| Brushing | Observational (10):  - Cohort (1)  - Case control (1)  - Cross-sectional (8) | Not serious | Serious | Not serious | Not serious |  | **(+)( )( )( )**  VERY LOW |
| **Interdental cleaning** | Observational (4):  - Cross-sectional (4) | Not serious | Not serious | Not serious | Not serious |  | **(+)(+)( )( )**  LOW |
| **Dental visits** | Observational (6):  - Cohort (2)  - Case control (1)  - Cross-sectional (3) | Not serious | Not serious | Not serious | Not serious | (-) Publication bias^5^ | **(+)( )( )( )**  VERY LOW |

***NOTE:***

***^1^*** ***Risk of bias****: The results of modified Newcastle-Ottawa Quality Assessment Scale were used for considered (see Table S2). Proportions of low, moderate and high risk of bias studies were 10:3:2, 6:4:0, 2:2:0 and 3:3:0 for fair and poor versus good OH, brushing, interdental cleaning and dental visit, respectively. From these, the numbers of low risk of bias studies were ≥ 50% for each pooling, hence, we graded them as “Not serious”.*

***^2^ Inconsistency:*** *Heterogeneity of all poolings were moderate to high level and might be problematic except interdental cleaning. Fortunately, sources of heterogeneity could be identified properly. Results from pooled effect of OH in “community-based studies” and pooled effect of dental visit among studies that clearly defined a regular dental visit as least once a year were quite consistent. While, the heterogeneity of brushing still had presented as moderate to high level after exploring possible sources (Table S8). Therefore, only brushing was considered as “Serious” for inconsistency, then the quality of evidence was downgraded to very low level.*

***^3, 4^ Large effect & Dose-response gradient:*** *The effect of OH on periodontitis was significant with the large pooled OR about 2 to 5 times. Moreover, it showed the dose-response relation, in other words, risk of periodontitis increased with worsening OH level (poor > fair > good OH). Therefore, the quality of evidence was upgraded from low to moderate level.*

***^5^*** ***Publication bias:*** *The publication bias was suspected in the pooled effect of dental visit from the Egger test (Table S9) and the contour enhanced-funnel plot (Figure S2-c). The quality of evidence was downgraded to very low level.*
